# Rare variant genetic landscape of familial chylomicronemia syndrome (FCS) in the United Kingdom

**DOI:** 10.1016/j.gimo.2025.103445

**Published:** 2025-07-14

**Authors:** Bilal Bashir, Natalie Forrester, Paul Downie, Sarah Marsh, Carolyn Dent, Anthony S. Wierzbicki, Charlotte Dawson, Jonathan Schofield, Fiona Jenkinson, Michael Mansfield, Dev Datta, Hannah Delaney, Yee Teoh, Paul Hamilton, Jaimini Cegla, Maryam Ferdousi, Anoushka Kamath, Pankaj Gupta, Ahai Luvai, Dawn O’Sullivan, Deanna Mamood, Jian Wang, Paul N. Durrington, Robert A. Hegele, Handrean Soran

**Affiliations:** 1 Faculty of Biology Medicine and Health, University of Manchester, United Kingdom; 2 Department of Endocrinology, Diabetes and Metabolism, Manchester University NHS Foundation Trust, Manchester, United Kingdom; 3 Bristol Genetics Laboratory, North Bristol NHS Trust, Bristol, United Kingdom; 4 Department of Clinical Biochemistry, Bristol Royal Infirmary, Bristol, United Kingdom; 5 Department of Metabolic Medicine and Chemical Pathology, Guy's and St. Thomas' Hospitals, London, United Kingdom; 6 Department of Diabetes, Endocrinology and Metabolism, Queen Elizabeth Hospital NHS Foundation Trust, Birmingham, United Kingdom; 7 Department of Clinical Biochemistry and Metabolic Medicine, Royal Victoria Infirmary, Newcastle upon Tyne, New Castle, United Kingdom; 8 Leeds Centre for Diabetes and Endocrinology, Leeds Teaching Hospitals NHS Trust, Leeds, United Kingdom; 9 Lipid Unit, University Hospital Llandough, Cardiff, United Kingdom; 10 Department of Clinical Chemistry, Sheffield Teaching Hospitals NHS Foundation Trust, Sheffield, United Kingdom; 11 Department of Chemical Pathology and Metabolic Medicine, Wrexham Maelor Hospital, Wrexham, United Kingdom; 12 Centre for Medical Education, Queen's University Belfast, Belfast, United Kingdom; 13 Division of Diabetes, Endocrinology and Metabolism, Imperial College London, London, United Kingdom; 14 University Hospitals of Leicester NHS Trust, Leicester, United Kingdom; 15 North of Scotland Genetics Laboratory, Polwarth Building, Aberdeen, Scotland, United Kingdom; 16 Department of Medical Genetics, School of Medicine, Medical Sciences and Nutrition, University of Aberdeen, Aberdeen, United Kingdom; 17 College of Medicine, University of Sulaimani, Sulaimani, Federal Region of Kurdistan, Iraq; 18 Robarts Research Institute, Western University, London, Ontario, Canada

**Keywords:** Autosomal recessive, Chylomicronemia, Genetic variants, Genetics, Hypertriglyceridemia

## Abstract

**Purpose:**

Familial chylomicronemia syndrome (FCS) is a rare autosomal recessive disorder. This study aimed to analyze the genotype distribution of FCS-causing genes in the United Kingdom.

**Methods:**

Data were anonymously collated from 2 genetic testing laboratories providing national genetic diagnosis services for severe hypertriglyceridemia in the United Kingdom.

**Results:**

As of December 2023, 880 individuals underwent genetic testing for FCS. The mean (SD) age at the time of genetic testing was 42.5 (15.3) years. After genotyping, 12.9% of the individuals (*n* = 114) received a genetic diagnosis of FCS. The detection rate of variant-positive multifactorial chylomicronemia syndrome, ie, heterozygous for pathogenic/likely pathogenic (P/LP) variants in 1 of the 5 canonical genes was 11.4% (*n* = 100).

Among 114 genetically proven FCS individuals, 52.6% (*n* = 60) had biallelic P/LP *LPL* variants (ie, LPL-FCS), 45.6% (*n* = 52) had biallelic non-*LPL* P/LP variants (ie, non-*LPL*-FCS) and 1.7% (*n* = 2) individuals were digenic. Among non-*LPL*-FCS (*n* = 52), the most common gene implicated was *GPIHBP1* (42.3%, *n* = 22), followed by *APOA5* (32.7%, *n* = 17), *LMF1* (13.5%, *n* = 7) and *APOC2* (11.5%, *n* = 6). Most variant-positive multifactorial chylomicronemia syndrome harbored P/LP variants in *LPL* (61%) or *APOA5* (37%).

The geographical distribution of FCS demonstrated regional variability, where the Northwest of England had the highest number of FCS cases per million population. Individuals of European geographic ancestry predominantly had LPL-FCS (60.9%); however, genotype was more diverse in individuals of non-European origin (*LPL* 47.1%, *GPIHBP1* 30.9%, *APOA5* 8.8%, *LMF1* 7.4%, and *APOC2* 4.4%). Variants in specific causal genes, *GPIHBP1* and *LMF1*, were predominantly observed in non-European FCS individuals.

**Conclusion:**

The genetic architecture of FCS in the United Kingdom is complex, with a substantial proportion affected by non-*LPL* FCS-causing genes. It also displays a significant regional and ethnic variations.

## Introduction

Chylomicronemia is the persistence of circulating chylomicrons after an overnight fast. Often it is accompanied by a constellation of symptoms, which is termed chylomicronemia syndrome (CS).^1^^,^^2^ It can manifest itself in type 3 dysbetalipoproteinemia, autoimmune hypertriglyceridemia, familial chylomicronemia syndrome (FCS), and multifactorial chylomicronemia syndrome (MCS). It is a rare metabolic disorder and, when accompanied by elevated triglyceride (TG) concentration beyond 10 mmol/L (885 mg/dL), puts an individual at risk of acute pancreatitis. FCS represents the distinctive form of CS. It is an autosomal recessive disorder caused by pathogenic variants in coding regions of genes encoding proteins involved in lipoprotein lipase (LPL) activity, including *LPL* (HGNC:6677) itself, and auxiliary genes, ie, *Apolipoprotein C2 (APOC2*; HGNC:609), *Apolipoprotein A5 (APOA5*; HGNC:17288), *Lipase maturation factor 1 (LMF1*; HGNC:14154), and *Glycosylphosphatidylinositol-anchored HDL-binding protein 1 (GPIHBP1*; HGNC:24945), responsible for optimal *LPL* functional activity.^3^^,^^4^

Historically, FCS was believed to be predominantly caused by variants in the *LPL* gene. Data representing clinical and biochemical characteristics of FCS individuals suggest that LPL-FCS represents over 80% of cases, and non-*LPL* gene variants are even rarer.^5^, ^6^, ^7^ Despite similarities in most of the clinical and biochemical characteristics, non-*LPL* FCS tends to respond better to conventional lipid-lowering therapy, exhibits less insulin resistance, has higher post-heparin *LPL* activity and higher levels of low-density lipoprotein cholesterol (LDL-C).^8^^,^^9^ We have recently compared the clinical and biochemical characteristics of individuals with *LPL* FCS and non-*LPL* FCS^9^ and validated FCS score in an ethnically diverse UK population.^10^ However, there are few data comparing the frequency and distribution of non-*LPL* FCS-causing genes and comparing clinical and biochemical characteristics of individuals between LPL-FCS and non-*LPL* FCS and between individual harboring non-*LPL* FCS gene variants.^8^

Prevalence estimates for FCS range widely from 1 in 100,000 to 1 in 1,000,000.^1^^,^^11^ The introduction of the FCS score by Moulin et al^2^ refines the clinical criteria for appropriate requesting of genetic tests and is validated for both *LPL*- and non-*LPL*-FCS. Non-*LPL*-FCS might have a relatively milder phenotype.^2^^,^^10^ Nevertheless, despite more judicious requests for genetic testing, FCS is likely underdiagnosed due to nonspecific signs and symptoms, and limited knowledge of the disease.^12^

Most cases of CS are polygenic and multifactorial, resulting from single pathogenic variants (variant-positive MCS) or are polygenic with or without secondary factors.^13^ Although FCS and MCS share clinical manifestations and are difficult to differentiate, MCS generally presents with a relatively milder phenotype, occurring at a relatively older age, and responds better to conventional lipid-lowering therapy. Previous studies have shown that MCS with heterozygous loss-of-function (LOF) variants in FCS-causing genes have an intermediate phenotype between FCS and variant negative MCS.^9^ Yet, the phenotype of MCS is highly heterogeneous, with numerous factors governing precise phenotype beyond the presence or absence of pathogenic variants and triglyceride levels.^14^^,^^15^

We have previously reported and compared the clinical and metabolic phenotypes of individuals with FCS and MCS in the United Kingdom.^9^ Using the same registry, we now aim to investigate the distribution and frequency of FCS-causing gene variants in the ethnically diverse UK population and explore their ethnic and geographical distribution. Additionally, we aim to report all P/LP variants and variants of uncertain significance (VUS) identified in the United Kingdom.

## Material and Methods

The data that support the findings of this study are available from the corresponding author upon request.

### Study participants and data collection

The UK FCS register was established in 2017 to study the natural history and unmet clinical needs of this rare disorder. The registry has been approved by the UK Health Research Authority (HRA) and Northwest-Greater Manchester South Research Ethics Committee to obtain genetic information from 6 regional lipid centers and 2 national genetics laboratories in the United Kingdom providing analysis for this disorder in the United Kingdom. Ethical approval was granted by the UK HRA and the Northwest-Greater Manchester South Research Ethics Committee (17/NW/0230).

Genetic analysis for all individuals was conducted at 2 accredited medical laboratories, ie, Bristol Genetics Laboratory (BGL) and Aberdeen Molecular Genetics Diagnostic Laboratory. These laboratories hold United Kingdom Accreditation Service with rigorous assessments conducted against the International Standard for medical laboratories.

We identified 880 individuals with CS from the United Kingdom who had been genotyped for FCS-causing genes up to December 2023. The UK National Health Service (NHS) necessitates individuals to have a TG concentration > 20 mmol/L (1770 mg/dL) and the absence of secondary factors for hypertriglyceridemia to be eligible for genetic testing for FCS. However, since 2018, with the introduction of the FCS Score*,* many centers have adopted a score of at least 8 as the threshold for eligibility for genetic testing.

Anonymous data were obtained retrospectively from both national laboratories. The geographical location of the participants was inferred to be the same as the origin of the request for genetic testing. We obtained information on sex and ethnicity from genetic test request forms, which typically include these demographic details as provided by the requesting clinician. In line with NHS England guidelines, ethnicity data are collected through an individual’s self-identification during health care interactions. Individuals are asked to select their ethnic group from standardized categories based on the Office for National Statistics census classifications. These categories are designed to capture a broad range of ethnic identities and are used consistently across NHS data collection systems.

In this study, we adopted this standardized approach, relying on the individual’s self-identified ethnicity as documented by the health care provider. When direct person’s input was unavailable, we depended on the referring clinician's assessment, acknowledging that this may introduce variability.

In this study, ethnicity was grouped into 2 categories:

1. European: This category predominantly included individuals of White European ancestry.
2. Non-European: Over 90% of individuals in this category were of South-Asian ancestry, whereas a minority of participants from other non-European backgrounds.

This study utilized data from a genetic testing laboratory, which focused on individuals meeting prespecified clinical criteria for genetic testing for severe hypertriglyceridemia. Because cascade testing or family screening is not routinely offered in the United Kingdom for this condition, the data set primarily includes individuals who were tested based on clinical indications and eligibility criteria defined by NHS alone. Therefore, the study does not have information regarding whether any of the cases are related and specific identification of index cases is not a key consideration within this cohort.

### Genetic analysis and pathogenicity ascertainment

The genetic analysis for suspected FCS individuals was performed at the BGL and the Aberdeen Molecular Genetics Diagnostic Laboratory. Details of the genetic testing have been explained elsewhere.^9^^,^^10^ Briefly, BGL used a custom-designed next generation sequencing (NGS) panel to test individuals for genes associated with FCS (National genomic test directory code R324, PanelApp version 1.2). The following genes were included in the analysis: *LPL* (HGNC:6677)*, GPIHBP1* (HGNC:24945)*, APOA5* (HGNC:17288)*, LMF1* (HGNC:14154)*,* and *APOC2* (HGNC:609). *CREB3L3* (HGNC:18855)*, GPD1* (HGNC:4455), and *APOE* (HGNC:613) genes were also included on this NGS panel. Details of the genes included on this panel can be accessed online at https://nhsgms-panelapp.genomicsengland.co.uk/. The Aberdeen laboratory performed Sanger sequencing of *LPL*, *GPIHBP1*, *APOA5*, *APOC2*, *LMF1*, and *APOE* genes. NGS data were analyzed on the Illumina NextSeq platform followed by sequence analysis using an open-source in-house pipeline with the hg19/GRCh37 human genome as a reference. Variant filtering was performed by registered clinical scientists using the bespoke in-house database. Sequence variants were analyzed and interpreted according to the American College of Medical Genetics and Genomics (ACMG) and Association of Clinical Genomic Sciences (ACGS) best practice guidance.^16^^,^^17^ The pathogenicity of the variants was also assessed using 2 online platforms. The Franklin tool by Genoox (https://franklin.genoox.com) uses the ACMG pathogenicity classification system, established in 2015, to interpret the pathogenicity of sequence variants. Franklin serves as a computerized implementation of these guidelines, originally designed for clinical assessment of variants. In addition, we used the online platform Varsome (https://varsome.com/). Varsome is a comprehensive variant interpretation platform^18^ that aggregates data from over 140 data sets, including but not limited to ClinVar,^19^ gnomAD,^20^ GWAS,^21^ and dbSNP,^22^ as well as proprietary databases and literature sources. It provides an interface to analyze and interpret genetic variants, offering detailed annotations, classification, and pathogenicity predictions. It uses sophisticated algorithms and expert-curated databases to facilitate accurate variant classification and interpretation as per ACMG guidelines. For variants with conflicting interpretations, manual confirmation of the pathogenicity of each variant using ACMG guidelines was done independently by 2 authors. Both of the commercially available tools provide automated variant classifications based on parameters set against the ACMG 2015 best practice guidance for variant classification. Although these tools are widely considered to help provide preliminary classifications, they are not to be used in isolation for diagnostic purposes. Our In-house variant classifications presented in this article are performed manually by registered clinical scientists in accredited laboratories, using a combination of resources. In UK laboratories, an additional set of variant classification guidelines (ACGS Best Practice Guidelines for Variant Classification in Rare Disease 2024)^16^ are adopted for supplementary use with the ACMG 2015 framework. The ACGS document reflects local UK practices and provides supporting guidance to apply codes in specific circumstances; however, these will not be in use by commercial US-based classifier tools and will account for a small degree of variance in classifications. Further variation is possible because of the manual nature of the classification process, which may incorporate a degree of professional judgement from the clinical scientists, particularly in rare scenarios, with less common variant types and in genes that are less well understood. Individuals with P/LP homozygous or compound heterozygous variants in 1 of the 5 canonical genes (*LPL*, *GPIHBP1*, *APOA5*, *APOC2*, and *LMF1*) received a genetic diagnosis of FCS. Rarer individuals with P/LP variants in 2 different genes (ie, double heterozygotes/digenic) also received a genetic diagnosis of FCS. Individuals with heterozygous P/LP variants, heterozygous P/LP pathogenic variants with an additional VUS, a heterozygous VUS, a homozygous VUS, or no pathogenic or VUS were classified as MCS.

All coding DNA (c.) variants described in this manuscript are based on the following reference sequences: *LPL*-NM_000237.3, *GPIHBP1*-NM_178172.6, *APOA5*–NM_052968.5, *LMF1*-NM_022773.4, and *APOC2*-NM_000483.5.

### Statistical analysis

Continuous variables are summarized as medians (interquartile range [IQR], Q1-Q3) for nonparametric data or mean (standard deviation [SD]) for parametric data and categorical variables are reported as percentages or frequencies. The χ^2^ test (or Fisher’s exact test) was used for categorical variables. Independent sample Mann-Whitney U test (nonparametric data) or independent sample *t* test (parametric data) was used to determine the difference between groups. A *P* value of <.05 was considered significant. Statistical analysis was carried out on SPSS (Version 25.0, IBM Corporation), and graphs were constructed using GraphPad Prism for Windows (Version 9.3.1).

## Results

### Prevalence and detection rate of FCS and variant-positive MCS

As of December 2023, a total of 880 individuals underwent genetic testing. Genetic analysis of the 5 canonical genes for FCS genes showed that 114 of 880 (12.9%) of clinically diagnosed individuals with CS were confirmed to have FCS. The detection rate of variant-positive MCS, ie, heterozygous for P/LP variants in 1 of the 5 canonical genes, was 100 in 880 (11.4%). In addition to this, 73 of 880 (8.3%) individuals had at least one VUS, either in homozygous, heterozygous, or compound heterozygous form. In individuals who are heterozygous for P/LP variants in 1 of the 5 canonical genes, an additional VUS was present in 11 of 100 (11%) individuals (Table 1).

**Table 1:** Detection rate of chylomicronemia syndrome

Diagnosis	Number of Participants (%) *n* = 880
FCS, no. (%)	114 (12.9)
MCS, no. (%) • P/LP variant-positive MCS, no. (%) • P/LP variant negative MCS, no. (%)	766 (87.1%) • 100 (11.4) • 666 (75.7)

Distribution of VUS in MCS Cohort (*n* = 766)	

MCS with only VUS	73 (9.5)
Variant-positive MCS with additional VUS	11 (11)

*FCS*, familial chylomicronemia syndrome, *MCS*, multifactorial chylomicronemia syndrome, *P/LP*, pathogenic/likely pathogenic, *VUS*, variant of uncertain significance.

**Figure 1:**
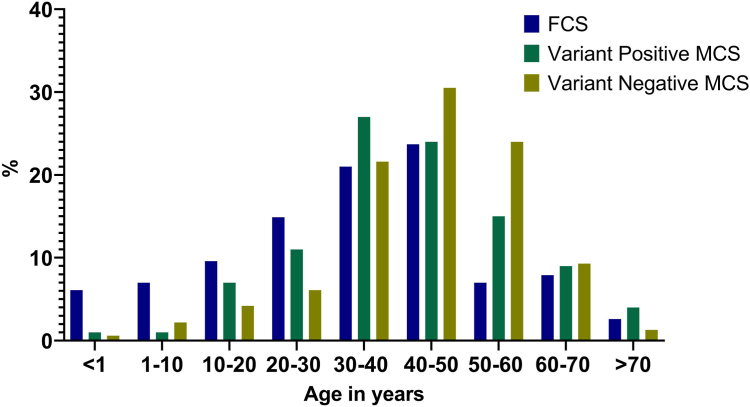
**Age distribution at genetic testing in each group.** FCS, Familial chylomicronemia syndrome; MCS, multifactorial chylomicronemia syndrome.

The mean (SD) age at the time of genetic testing was 42.5 (15.3) years. Among all, 60.8% (*n* = 535) of tested individuals were males, 35.8% (*n* = 315) were females, whereas sex was not known for 3.4% (*n* = 30). A total of 1.4% (*n* = 12) individuals were tested in infancy, and among them, 58.3% (*n* = 7) were genetically confirmed to have FCS, and 4.2 % (*n* = 37) were tested before the age of 10 years, and among them, 40.5% (*n* = 15) were genetically confirmed to have FCS. The proportion of individuals tested stratified according to age is summarized in Figure 1, Supplemental Table 1. The mean age at genetic testing was younger in females (40.2 [16.3] vs 44.0 [14.3] years, *P* < .001), and there was a nonsignificant trend of genetic diagnosis of FCS at an earlier age in females (32.2 [17.5] vs 38.1 [19.1] years, *P* < .1; *P* = .09). Although there was no difference in the sex distribution in individuals diagnosed with FCS (females 47.4% [*n* = 54], males 51.8% [*n* = 59], and unknown 0.9% [*n* = 1]), a significantly greater proportion of females who were tested received a genetic diagnosis of FCS compared with males (17.1% [*n* = 54/315] vs 11.0% [*n* = 59/535], *P* = .011).

### Genetic spectrum of FCS and variant-positive MCS

**Figure 2:**
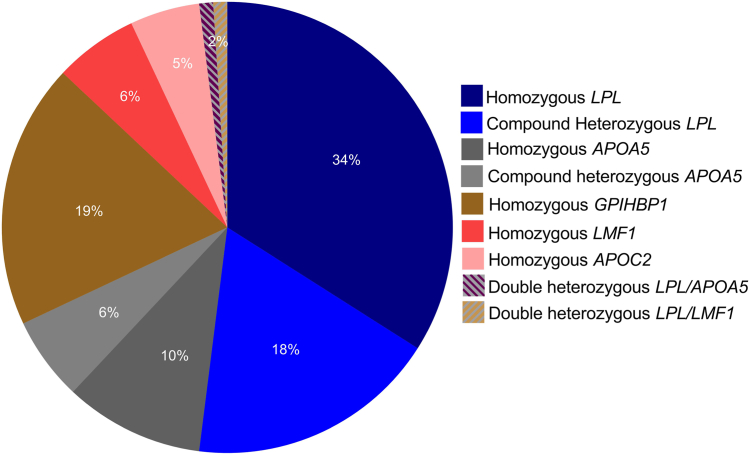
**Genetic spectrum of FCS and relative proportion of individual genotypes in the whole FCS cohort.** FCS, Familial chylomicronemia syndrome.

Among 114 genetically proven FCS individuals, 52.6% (*n* = 60) had biallelic P/LP *LPL* variants (ie, LPL-FCS), 45.6% (*n* = 52) had biallelic non-*LPL* P/LP variants (ie, non-*LPL*-FCS), and 1.7% (*n* = 2) had variants in 2 different canonical genes (double heterozygous/digenic). Genotype frequencies of *LPL*- and non-*LPL*-FCS are summarized in Figure 2.

Among LPL-FCS individuals, 65.0% (*n* = 39) were homozygous, and 35.0% (*n* = 21) carried P/LP *LPL* variants in compound heterozygous form. The most common pathogenic *LPL* variant identified in this cohort was c.644G>A p.(Gly215Glu) missense variant, which was present either in homozygous or heterozygous form in 41.7% (*n* = 25) LPL-FCS individuals. 6.7% (*n* = 4) of LPL-FCS individuals were reported to have whole gene deletion (1 homozygous, 3 heterozygous, 2 with c.644G>A p.(Gly215Glu) missense variant in the other allele, and 1 with c.721C>T p.(Pro241Ser) missense variant in the other allele).

Among non-*LPL*-FCS (*n* = 52), the most common gene implicated was *GPIHBP1*, which constituted 42.3% (*n* = 22) of non-*LPL*-FCS cohort. All the participants with LOF variants in *GPIHBP1* were homozygous for respective P/LP variants. One participant homozygous for exon 3 and 4 deletion had an additional *LPL* pathogenic variant (c.553G>A p.(Ala185Thr)). The most frequent variant identified in this cohort of FCS with *GPIHBP1* variants was homozygous exon 3 and 4 deletion, accounting for 68.2% (*n* = 15) of *the GPIHBP1* variant cohort. This was followed by *APOA5*, constituting 32.7% (*n* = 17) of non-*LPL*-FCS, of whom the majority were homozygous (64.7%, *n* = 11). The most frequent variant identified was the nonsense variant in c.289C>T p.(Gln97∗) (*n* = 7), most of them (*n* = 5) in compound heterozygous form and frameshift variant c.427del p. (Arg143Alafs∗57) in homozygous form (*n* = 5). *LMF1* and *APOC2* constituted the remainder of the non-*LPL*-FCS cohort (13.5% [*n* = 7] and 11.5% [*n* = 6], respectively). All of them were in a homozygous form in which the most frequent variants identified for *LMF1* were nonsense variants in c.1024C>T p.(Arg342∗) and c.1264C>T p.(Gln422∗) (43% [*n* = 3] each). Similarly, for *APOC2*, all of the FCS individuals were homozygous, and the most frequent variants identified were c.215+2del (p.?) and c.215G>C p.(Arg72Thr) (33%, *n* = 2 each). In addition, 2 digenic FCS individuals were identified, one harboring P/LP variants in *LPL* (c.347G>A p.(Arg116Glu)) and *LMF1* (c.1348A>T (p.Arg450∗)) with the other having P/LP variant in *LPL* (c.644G>A p.(Gly215Glu)) and *APOA5* (c.289C>T p.(Gln97∗)). A summary of the most common variants detected in each of the canonical genes is shown in Table 2. Further details, including the relative frequency and classification of the complete set of variants identified in this study can be found in Supplemental Tables 2-4.

**Table 2:** Most common variants in each of the canonical genes

Gene Symbol	HGNC ID	RefSeq	HGVS c.description	HGVS p.description	HGVS g.description (GRCh37/hg19 Genome Assembly)	ACMG Classification and Score^a^
**FCS**						
*LPL*	6677	NM_000237.3	c.644G>A	p.(Gly215Glu)	NC_000008.10:g.19811733G>A	P (PS3, PM3_STR, PM5, PP3, PP4)
*APOA5*	17288	NM_052968.5	c.289C>T	p.(Gln97∗)	NC_000011.9:g.116661656G>A	P (PVS1_STR, PM3_STR)
*GPIHBP1*	24945	NM_178172.6	Deletion of exon 3-4	p.?	NC 000008.10:g.(?_144296868)_(144298897_?)del	P (PVS1, PM2, PM3)
*LMF1*	14154	NM_022773.4	c.1024C>T	p.(Arg342∗)	NC_000016.9:g.921215G>A	P (PVS1, PM2, PM3, PP4)
*LMF1*	14154	NM_022773.4	c.1264C>T	p.(Gln422∗)	NC_000016.9:g.920035G>A	P (PVS1, PM2, PM3)
*APOC2*	609	NM_000483.5	c.215+2del	p.?	NC_000019.9:g.45452119del	LP (PVS1_STR, PM2, PM3_SUP, PP4)
*APOC2*	609	NM_000483.5	c.215G>C	p.(Arg72Thr)	NC_000019.9:g.45452117G>C	LP (PM1_SUP, PM2, PM3_SUP, PP3, PP4)
**Variant-positive MCS**						
*LPL*	6677	NM_000237.3	c.644G>A	p.(Gly215Glu)	NC_000008.10:g.19811733G>A	P (PS3,PM3_STR, PM5, PP3, PP4)
*APOA5*	17288	NM_052968.5	c.289C>T	p.(Gln97∗)	NC_000011.9:g.116661656G>A	P (PVS1_STR, PM3_STR)
*GPIHBP1*	24945	NM_178172.6	c.394C>T	p.(Gln132∗)	NC_000008.10:g.144297232C>T	LP (PVS1_STR, PM2, PM3_MOD, PP4)
*LMF1*	14154	NM_022773.4	c.244_245del	p.(Arg82Glyfs∗81)	NC_000016.9:g.1004615_1004616del	P (PVS1, PM2, PM3_SUP, PP4)
**VUS**						
*LPL*	6677	NM_000237.3	c.953A>G	p.(Asn318Ser)	NC_000008.10:g.19813529A>G	VUS (PS3_STR, PP1_MOD, BA1_VSTR, BP4)
*APOA5*	17288	NM_052968.5	c.553G>T	p.(Gly185Cys)	NC_000011.9:g.116661392C>A	VUS (BP6_SUPP)
*GPIHBP1*	24945	NM_178172.6	c.484G>A	p.(Glu162Lys)	NC_000008.10:g.144297322G>A	VUS (PM2_MOD, BP4_SUPP)
*GPIHBP1*	24945	NM_178172.6	c.52+28G>A	p.?	NC_000008.10:g.144295222G>A	VUS (PM2_MOD)
*GPIHBP1*	24945	NM_178172.6	c.416C>T	p.(Pro139Leu)	NC_000008.10:g.144297254C>T	VUS (PM2_MOD)
*GPIHBP1*	24945	NM_178172.6	c.188T>C	p.(Leu63Pro)	NC_000008.10:g.144296894T>C	VUS (PM2_MOD)
*LMF1*	14154	NM_022773.4	c.683G>A	p.(Gly228Glu)	NC_000016.9:g.943053C>T	VUS (PM2_SUPP, BP1_SUPP)
*LMF1*	14154	NM_022773.4	c.1247G>A	p.(Arg416Gln)	NC_000016.9:g.920052C>T	VUS (PM2, PM3_SUP, PP3, PP4)

*ACMG*, American College of Medical Genetics and Genomics; *APOA5*, apolipoprotein A5; *APOC2*, apolipoprotein C2; *FCS*, familial chylomicronemia syndrome; *GPIHBP1*, glycosylphosphatidylinositol-anchored high-density lipoprotein-binding protein 1; *HGVS*, Human Genome Variation Society; *LMF1*, lipase maturation factor 1; *LP*, likely pathogenic; *LPL*, lipoprotein lipase; *MCS*, multifactorial chylomicronemia syndrome; *P*, pathogenic; *VUS*, variant of uncertain significance.

a ACMG scoring can vary slightly between platforms because of different rule implementations and availability of clinical data.

Of all tested individuals, 87.1% (*n* = 766) did not meet the genetic criteria for FCS. Among them, 87.0% (*n* = 666) did not harbor P/LP gene variants in any of the FCS-causing genes. Among MCS individuals, 13.0% (*n* = 100) harbored P/LP gene variants in one of the canonical genes in the heterozygous form, and 9.5% (*n* = 73) had VUS in the homozygous, heterozygous, double heterozygous/digenic, or compound heterozygous form. Among MCS with heterozygous P/LP gene variants, 11.0% (*n* = 11) had an additional VUS. Similar to FCS, the majority of variant-positive MCS individuals harbored P/LP variants in the *LPL* gene (61.0%, *n* = 61). This was followed by P/LP variants in *APOA5* (37.0%, *n* = 37). A small minority had P/LP heterozygous variants in *LMF1* and *GPIHBP1* (1%, *n* = 1 each). Similar to LPL-FCS individuals, among the variant-positive LPL-MCS individuals, the c.644G>A p.(Gly215Glu) variant was the most prevalent (37.7%, *n* = 23). In *APOA5* variant-positive MCS individuals, c.289C>T p.(Gln97∗) was most prevalent (32.4%, *n* = 12) followed by c.823C>T p.(Gln275∗) (27.0%, *n* = 10). There was a single case each of variant-positive MCS with P/LP variant in *GPIHBP1* (c.394C>T p.(Gln132∗)) and *LMF1* (c.244_245del p.(Arg82Glyfs∗81)).

### Clinical features of FCS cohort stratified according to genotype

Table 3 summarizes key clinical and demographic characteristics of FCS individuals with different gene variants. The median (IQR) age at genetic testing and hence molecular confirmation was 36.8 (22.8-46.3) years, with no significant difference between each of the genotypes (*P* = .3). Parental consanguinity and non-European ancestry were more prevalent in FCS with *GPIHBP1* and *LMF1* variants. There was a progressive delay from the initial manifestation of symptoms to molecular confirmation of several years between age at symptom onset (17.5 [6.2-27.0] years), age at clinical diagnosis (24.0 [10.0-35.5] years), and age at genetic confirmation (36.8 [22.8-46.3] years) of FCS that was consistent across all subgroups (Table 3).

**Table 3:** Clinical characteristics of FCS cohort

Variable	All	*LPL*	*APOA5*	*GPIHBP1*	*LMF1*	*APOC2*	Digenic
Age^a^ (median-IQR), years	36.8 (22.8-46.3)	36.3 (23.2-47.4)	35.2 (17.4-46.0)	36.0 (18.8-42.1)	41.0 (29-65.0)	39.0 (17.3-54.4)	48 and 44
Sex^b^							
Females, no. (%)	54 (48)	30 (50)	9 (53)	8 (38)	3 (43)	2 (33)	2 (100)
Males, no. (%)	59 (52)	30 (50)	8 (47)	13 (62)	4 (57)	4 (67)	0 (0)
Ethnicities^a^							
European, no. (%)	46 (40)	28 (47)	11 (65)	1 (4)	2 (29)	3 (50)	1 (50)
Non-European, no. (%)	68 (60)	32 (53)	6 (35)	21 (95)	5 (71)	3 (50)	1 (50)
Parental consanguinity^c^, no. (%)	41 (48)	16 (36)	4 (36)	13 (77)	6 (85)	1 (33)	1 (50)
Asymptomatic^c^, no. (%)	5 (6)	1 (2)	1 (9)	0 (0)	3 (43)	0 (0)	0 (0)
Age at symptoms^c^ (median-IQR), years	17.5 (6.2-27.0)	13.5 (3.5-27.5)	16.5 (9.0-30.7)	21.0 (6.0-26.0)	19.0 (9.5-27.0)	1, 30 and 7^e^	19 and 30^e^
							
Age at clinical diagnosis^c^ (median-IQR), years	24.0 (10.0-35.5)	18.0 (5.0-34.5)	30.0 (19.0-35.0)	24.0 (3.5-33.5)	26.0 (18.0-39.0)	32,42 and 7^e^	48 and 44^e^
Acute Pancreatitis^c^, no. (%)	69 (81)	38 (84)	10 (91)	14 (82)	3 (43)	2 (67)	2 (100)
Recurrent Acute Pancreatitis^c^, no. (%)	64 (93)	36 (95)	8 (80)	14 (100)	3 (100)	2 (100)	1 (50)
Unexplained abdominal pain^c^, no. (%)	66 (78)	38 (85)	8 (73)	14 (82)	3 (43)	2 (67)	1 (50)
Age at pancreatitis^c^ (median-IQR), years	24 (11.5-31.0)	26 (10.7-33.0)	18.5 (9.0-33.5)	24.0 (14.2-27.0)	24.0 (22.0-31.0)	1 and 30^e^	19 and 36^e^
BMI^c^ (median-IQR)-kg/m^2^	24.5 (20.6-26.9)	24.3 (20.9-26.3)	26.8 (23.6-28.2)	23.9 (20.6-25.5)	26.4 (20.6-27.2)	19.6, 26.3 and 20.3^e^	33.8 and 31.9^e^
Diabetes Mellitus^c^, no. (%)	19 (22)	11 (24)	2 (18)	3 (18)	1 (14)	1 (33)	1 (50)
Hypertension^c^, no. (%)	12 (14)	7 (16)	2 (18)	1 (6)	0 (0)	1 (33)	1 (50)
CVD^c^, no. (%)							
IHD	2 (2)	2 (4)	0 (0)	0 (0)	0 (0)	0 (0)	0 (0)
PVD	1 (1)	1 (2)					
CVA/TIA	1 (1)	1 (2)					
Peak TG^c^^,^^d^ (median-IQR)-mmol/L	42.4 (31.8-57.6)	46.2 (31.4-60.8)	36.0 (31.4-41.7)	49.4 (38.8-58.5)	44.8 (20.7-53.7)	63.3, 42.0 and 21.0^e^	39.0 and 64,2^e^
Trough TG^c^^,^^d^ (median-IQR)-mmol/L	7.2 (4.0-12.5)	7.5 (4.1-12.6)	9.2 (3.1-13.4)	6.3 (4.7-13.4)	6.6 (3.9-12.4)	8.3, 9.0 and 4.0^e^	1.9 and 2.6^e^
Medication^c^							
Fibrates	46 (54)	23 (52)	8 (20)	8 (47)	5 (71)	2 (67)	2 (100)
Statins	39 (46)	15 (35)	6 (60)	9 (53)	5 (71)	2 (67)	2 (100)
Omega-3 FA	26 (32)	12 (27)	3 (30)	4 (23)	5 (71)	1 (33)	1 (50)
Volanesorsen	26 (31)	14 (32)	5 (50)	6 (35)	1 (14)	0 (0)	0 (0)
Ezetimibe	5 (6)	4 (9)	0 (0)	1 (6)	0 (0)	0 (0)	0 (0)
Response to LLT^c^^,^^f^– no. (%)	24 (28)	8 (18)	2 (18)	6 (35)	4 (57)	2 (67)	2 (100)
TG < 2mmol/L^c^, no. (%)	3 (3)	1 (2)	1 (9)	0 (0)	0 (0)	0 (0)	1 (50)

*BMI*, body mass index; *CVA*, cerebrovascular accident; *CVD*, cardiovascular disease; *IHD*, ischemic heart disease; *IQR*, interquartile range; *LLT*, lipid-lowering therapy; *PVD*, peripheral vascular disease; *TG*, triglyceride; *TIA*, transient ischemic attack.

a Data available for all 114 FCS cases, age the time of genetic testing, ie, genetic confirmation of FCS.

b Data available for 113 FCS cases.

c Data available for 85 FCS cases; 45 LPL-FCS, 11 *APOA5*-FCS, 17 *GPIHBP1*-FCS, 7 LMF-FCS, 3 *APOC2*-FCS, and 2 Digenic FCS.

d Peak TG refers to the highest TG concentration recorded in an individual’s history at the time of data capture, whereas Trough TG denotes the lowest recorded TG concentration.

e Individual figures reported because of ≤3 cases in this group.

f Reduction in TG >20% from baseline (TG concentration at the time of LLT initiation) after the introduction of conventional LLT, ie, statins, fibrates, or O3FA.

### Molecular changes in the FCS cohort

In a cohort of 114 participants with FCS, encompassing 230 variants, we identified 56 unique variants in this cohort. Of these, 35 were *LPL* variants, and 21 were non-*LPL* variants. Missense and nonsense variants constituted most of the genetic alterations. This included 21 novel variants (13 in *LPL*, 4 in *APOA5*, 2 in *LMF1*, and 1 each in *GPIHBP1* and *APOC2*) (Supplemental Tables 2 and 3). Missense variants accounts for 44.6% (*n* = 25) and nonsense variants 21.4% (*n* = 12). The remaining comprised splice site variants (12.5%, *n* = 7), frameshift variants (14.3%, *n* = 8), large deletions (5.3%, *n* = 3), and a partial exon duplication (1.8% *n* = 1). Among *LPL* variants, 60.0% (*n* = 21) were missense, and 14.3% (*n* = 5) were nonsense variants. In contrast, non-*LPL* variants demonstrated greater molecular diversity, with 33.3% (*n* = 7) being nonsense, whereas splicing, frameshift, and missense constituted 19.0% (*n* = 4) each (Supplemental Figure 1).

### Variants of uncertain significance

In addition to P/LP variants in FCS and variant-positive MCS individuals, 10.9% (*n* = 73) of individuals harbored 1 or more VUS in one of the canonical genes. Most of the individuals with VUS had *LPL* variants (43.8%, *n* = 32), followed by *APOA5* (31.5%, *n* = 23), *LMF1* (19.2%, *n* = 14) and *GPIHBP1* (5.5%, *n* = 4). The most common VUS in *LPL* was c.953A>G p.(Asn318Ser) found in 53.5% (*n* = 17) of individuals harboring *LPL* VUS. The most common *APOA5* VUS was c.553G>T p.(Gly185Cys), detected in 43.5% (*n* = 10) of individuals with an *APOA5* VUS, and in *LMF1* it was c.683G>A p.(Gly228Glu) and c.1247G>A p.(Arg416Gln) (14.3%, *n* = 2 each). VUS in *GPIHBP1* included 1 each of c.484G>A p.(Glu162Lys), c.52+28G>A p.?, c.416C>T p.(Pro139Leu), and c.188T>C p.(Leu63Pro) (Supplemental Table 4). The majority of individuals harbored heterozygous VUS; however, a small proportion of individuals (17.8%, *n* = 13) had a VUS in the homozygous state (*LPL* 46.1% [*n* = 6], *APOA5* 7.7% [*n* = 1], *LMF1* 38.5% [*n* = 5], *GPIHBP1* 7.7%, [*n* = 1]).

### Sex differences in FCS and variant-positive MCS

There was no difference in gender distribution between LPL-FCS and non-*LPL*-FCS: for LPL-FCS; females vs males: 50% (*n* = 30) vs 50% (*n* = 30), for non-*LPL*-FCS; females vs males: 45.2% (*n* = 24) vs 54.7% (*n* = 29) *P* = .6]. In FCS, the mean age (SD) of genetic testing was 35.0 (18.7) years and was numerically but not statistically significantly younger in females compared with males (32.2 [17.5] vs 38.1 [19.1] years, *P* < .1; *P* = .09). Out of all FCS individuals tested in infancy, 71.4% (*n* = 5) were females. Similarly, in variant-positive MCS, there was no difference in gender distribution between *LPL-* and non-*LPL* heterozygotes (*LPL*-variant-positive MCS; females vs males: 41.7% [*n* = 25] vs 58.3% [*n* = 35], non-*LPL*-variant-positive MCS; females vs males: 43.5% [*n* = 17] vs 56.4% [*n* = 22] *P* = .8); however, in variant-positive MCS cohort, age (SD) at time of genetic testing was younger in females compared with males (34.8 [15.6] vs 46.2 [15.1] years, *P* < .001).

### Regional variability in FCS prevalence and detection rates across the United Kingdom

**Figure 3:**
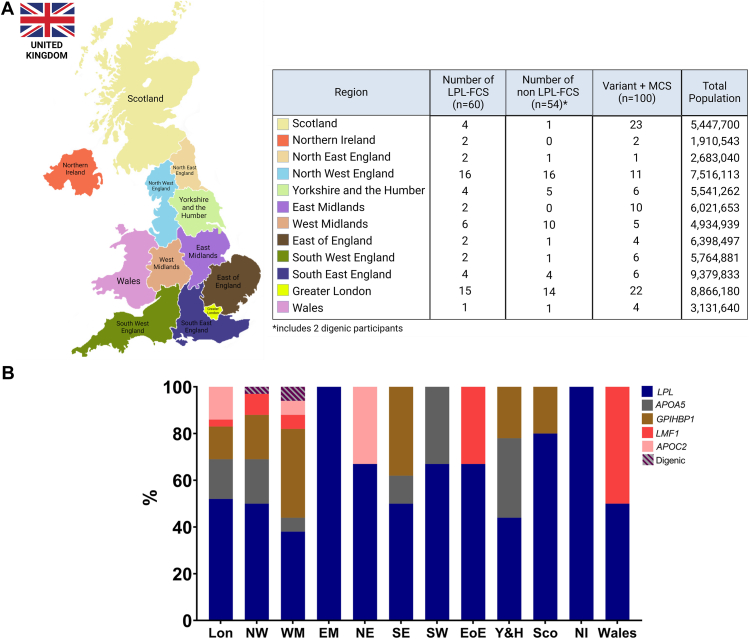
**Geographical distribution of FCS cases.** A. The geographical distribution of LPL-FCS, non*-*PL-FCS, and variant-Positive MCS across the United Kingdom along with the total population of the region. B. Distribution of individual genotypes across different regions. FCS, Familial chylomicronemia syndrome; LPL, lipoprotein lipase; MCS, multifactorial chylomicronemia syndrome; EM, East Midlands; EoE, East of England; Ire, Ireland; Lon, London; NI, Northern Ireland; NE, Northeast; NW, Northwest; Sco, Scotland; SE, Southeast; SW, Southwest; WM, West Midlands; Y&H, Yorkshire & Humber.

The geographical distribution of FCS in the United Kingdom demonstrates significant regional variability, with marked differences in prevalence among individuals possessing *LPL* genotypes compared with those with non-*LPL* genotypes (Figure 3). Regional disparities were also evident in both the frequency of FCS testing and the corresponding detection rates. Scotland recorded the highest testing rate at 30.8 tests per million population; however, the detection rate for FCS was comparatively low at 3.0%. Conversely, the Northwest of England achieved the highest detection rate (29.3%) despite conducting fewer tests per million population (14.5). London exhibited a moderate testing rate (20.0 per million) and a relatively high detection rate of 16.2%. East Midlands and Southwest demonstrated low detection rates (2.6% and 3.4%, respectively), despite moderate testing frequency (Supplemental Table 5).

**Figure 4:**
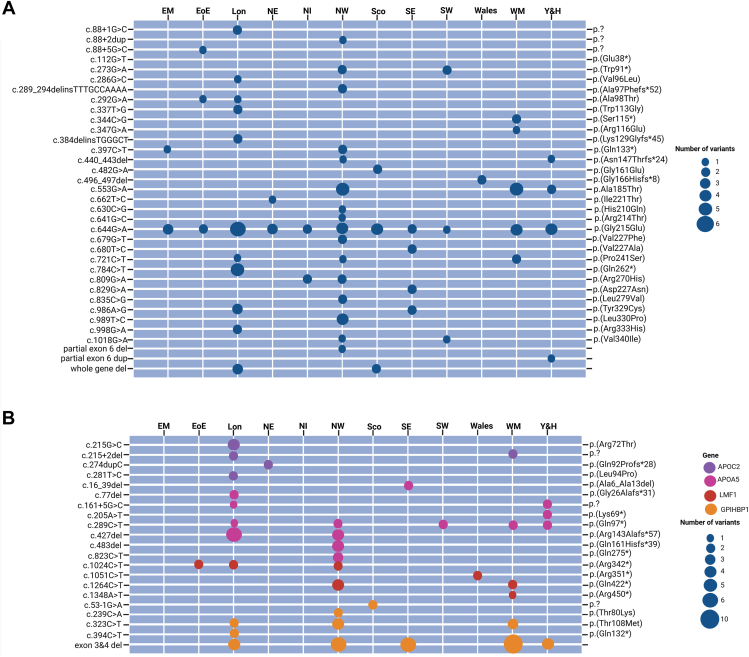
**Distribution of each FCS gene variant in United Kingdom.** The relative proportion of individual P/LP gene variants across different regions of the United Kingdom for LPL-FCS (A) and non-*LPL*-FCS (B). FCS, Familial chylomicronemia syndrome; LPL, lipoprotein lipase; P/LP, pathogenic/likely pathogenic. EM, East Midlands; EoE, East of England; Ire, Ireland; Lon, London; NI, Northern Ireland; NE, North East; NW, North West; Sco, Scotland; SE, South East; SW, South West; WM, West Midlands; Y&H, Yorkshire & Humber.

The Northwest of England manifests the highest number of FCS cases, accounting for 28.1% (*n* = 32) of the total FCS cohort, with an equal proportion of *LPL*- and non-*LPL*-FCS. London comprises 25.4% (*n* = 29) of the total FCS cohort within the United Kingdom. The proportion of LPL-FCS was slightly higher compared with non-*LPL*-FCS individuals: (51.7% [*n* = 15] vs 48.3% [*n* = 14]). The West Midlands region accounted for 14.0% (*n* = 16) of the total FCS cases; however, the proportion of LPL-FCS is comparatively lower than that of non-*LPL*-FCS (37.5% [*n* = 6] vs 62.5% [*n* = 10]). Based on the number of diagnosed cases, the prevalence of FCS in England was 2 per million. Scotland, Wales, and Northern Ireland together had 8.0% (*n* = 9) cases of FCS with predominance of LPL-FCS (77.8%, *n* = 7) compared with non-*LPL*-FCS (22.2%, *n* = 2). The prevalence of disease was approximately 1 per million in Scotland, Northern Ireland, and Wales. The regional distribution of individuals diagnosed with FCS, alongside those with variant-positive MCS and the total population of the region, is summarized in Figure 3A and B and the distribution of individual variants in individuals with FCS across the United Kingdom in Figure 4.

### Ethnic variations in genotype in FCS

**Figure 5:**
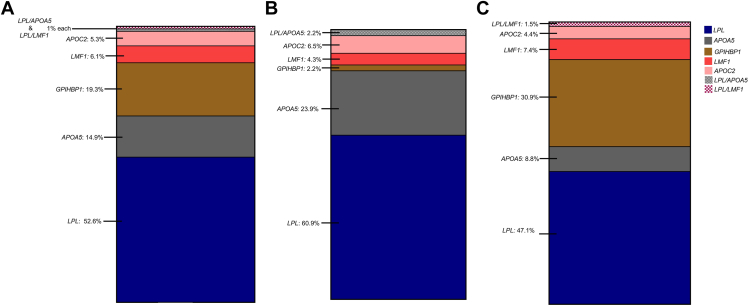
**Ethnic distribution of FCS-causing genes.** Relative proportion of individual genotypes in whole cohort (A), European FCS (B), and non-European FCS (C). FCS, Familial chylomicronemia syndrome.

When stratified based on ethnicity, individuals of non-European ethnic origin comprised 59.6% (*n* = 68) of the FCS cohort. There was a trend of early age of diagnosis in non-European FCS individuals (39.0 [22.2] vs 32.3 [15.4], *P <* .1; *P* = .08). There was no difference in sex distribution between both groups (*P* = .44). Individuals of non-European ethnic origin predominantly exhibited non-*LPL* gene variants. Conversely, participants of European origin primarily presented variants in the *LPL* gene. Notably, variants in specific causal genes, *GPIHBP1* and *LMF1*, were predominantly observed in non-European FCS individuals. A comprehensive summary of the spectrum of pathogenic variants in the European and non-European FCS cohort is presented in Figure 5.

## Discussion

In this study, we report the presence of rare variants within 5 canonical genes associated with FCS among all individuals who have undergone genetic diagnosis for severe hypertriglyceridemia in the United Kingdom. Among the 880 samples analyzed, 12.9% met the genetic diagnostic criteria for FCS. Additionally, 11.4% of the tested individuals displayed heterozygous LOF variants in one of the canonical genes, consistent with MCS. This molecular analysis represents the largest, most diverse and most novel data set of FCS and variant-positive MCS individuals reported to date. Encompassing data from the only laboratories offering genetic analysis for severe hypertriglyceridemia in the United Kingdom, this data set comprehensively encapsulates all individuals with a molecular diagnosis of FCS in the United Kingdom.

Our study delineates several significant and novel findings, notably the prevalence disparity between *LPL* and non-*LPL* forms of FCS, variability in disease prevalence across geographical regions in the United Kingdom, diverse molecular changes in non-*LPL*-FCS-causing genes, genetic diversity contingent upon the ethnic origins of FCS individuals, and the relative scarcity of certain variants, such as *GPIHBP1* and *LMF1*, among individuals of European descent compared with their non-European counterparts.

The prevalence of FCS has been a topic of active debate, with estimates ranging between 1 in 100,000 to 1 in 1,000,000.^11^ We identified 114 cases of FCS in England, corresponding to an approximate prevalence of 2 per million individuals.^23^ The number of FCS individuals in Scotland was 5, and Wales and Northern Ireland (NI) had 2 cases each corresponding to an estimated prevalence of 1 per million each. However, with the wider availability of genetic testing and introduction and validation of the FCS score^2^^,^^10^ to allow better selection of individuals for genetic testing, the number of cases diagnosed with FCS and hence the prevalence of FCS and variant-positive MCS is likely to increase. The estimated prevalence of FCS also varied according to the geographical area of the country where Northwest of England had the highest number of FCS cases per million followed by London and West Midlands. In this report and our previously published FCS registry data,^9^ we observed that a relatively higher proportion of individuals from ethnic minorities, specifically individuals of South-Asian descent have received a molecular diagnosis of FCS.

Consanguineous unions are relatively common in certain ethnic minority groups, particularly individuals of South-Asian descent,^24^ who constituted a considerable proportion of this cohort. Consanguineous marriages are associated with a 2.9-fold increased risk of developing autosomal recessive disorders, which may explain the relatively higher proportion of FCS participants in this ethnicity group.^25^ This might also explain the relatively higher prevalence of disease in certain geographical regions because the proportion of ethnic minorities, particularly descendants of South-Asian origin, in London, West Midlands, and Northwest, particularly Greater Manchester,^26^ is relatively higher compared with other areas.^27^ This observation presents a unique opportunity to enhance regional genetic services in regions where consanguineous unions are common. By educating families about autosomal recessive disorders, we can empower them to make informed reproductive choices and reduce genetic risks. Implementing effective communication tools and providing timely, high-quality genetic counseling are crucial strategies for achieving these goals.^28^

The prevalence of non-*LPL* variants was higher in our cohort compared with earlier observations in other cohorts.^5^, ^6^, ^7^ Complementing our previous observations, there was a greater heterogeneity in the genetic makeup of the non-European FCS cohort, in which more than half of the non-European FCS individuals had non-*LPL* FCS variants, most commonly *GPIHBP1*, which was scarce in the European cohort. In our whole cohort, there were 35 unique P/LP *LPL* variants identified. The most common *LPL* variant identified was the c.644G>A p. (Gly215Glu) missense variant that was reported in 16.6% (*n* = 10) of LPL-FCS individuals in homozygous state, 25% (*n* = 15) in compound heterozygous state. This variant was also reported as the most common LPL-FCS-causing variant in a cohort from the Spanish dyslipidemia registry^7^ and second most common in a cohort of FCS from Canada, where the most common *LPL* variant was the c.701C>T p.(Pro234Leu) missense variant^5^ that was only observed in 2 MCS individuals in heterozygous state in our cohort. Among non-*LPL*-FCS, 21 P/LP variants were identified, with the c.289C>T p.(Gln97∗) nonsense variant being the most common, reported in 41.1% (*n* = 7) *APOA5* FCS individuals. Similarly, an exon 3 and 4 deletion was seen in 68% (*n* = 15) of participants with *GPIHBP1* gene-related FCS. Nonsense variants in c.1264C>T p.(Gln422∗), c.1024C>T p.(Arg342∗) in the *LMF1* gene constituted 42.8% (*n* = 3 each) for *LMF1* related FCS. In addition to this, we identified 43 VUS, 14 in *LPL*, 11 in *APOA5*, 14 in *LMF1*, and 4 in *GPIHBP1*. VUS in homozygous state were present in 6 individuals in *LPL*, 1 in *APOA5*, 5 in *LMF1*, and 1 in *GPIHBP1*.

The detection rate of P/LP variants varies depending on the population, as well as the screening method applied. Historically guidance from the NHS national genomic test directories for Scotland and England mandated the elevation of triglycerides > 20 mmol/L (1770 mg/dL) in the absence or well-controlled secondary factors. However, since the introduction of the FCS score by Moulin et al^2^ many clinicians have adapted to a score of ≥8 as a referral criterion. Although we have validated this score and have demonstrated that the detection rate of FCS is significantly better with the use of the score,^10^ a significant proportion of the cohort reported predates the introduction of the score, and additionally, there exists the regional variations in thresholds and criteria used to select individuals for genetic testing. The observed discrepancies in testing frequency and FCS detection rates across regions may stem from variations in the diagnostic thresholds used by clinicians, such as the broad criteria in the National Genomic Test Directory^29^ vs stricter scores, such as the FCS score.^2^^,^^10^ Differences in clinician awareness,^12^ referral patterns, and access to genetic testing services may also contribute. Additionally, regional disparities in health care resources and population characteristics could influence both testing intensity and detection outcomes. The detection rate of FCS in this whole cohort of 880 individuals was 12.9%, which was comparable to the detection rate of FCS in the Spanish dyslipidemia registry (26/238, 10.9%)^7^ and a Canadian cohort of individuals with normoglycemia and severe hypertriglyceridemia and of European geographic ancestry (12/110, 10.9%).^30^ Similarly, the detection rate of LPL-FCS in a cohort of the Italian population has been reported at 11.8% and variant-positive MCS at 8.2%.^31^ The detection rate was similar despite the different thresholds applied to define severe hypertriglyceridemia, ie, >20 mmol/L (>1770 mg/dL) in our cohort, whereas >1000 mg/dL (11.2 mmol/L) in other cohorts. In contrast, it was significantly higher in an Italian cohort (12/32, 37.5%), in which the inclusion criteria stipulated having TG>1000 mg/dL (11.2 mmol/L) after 6 months of maximum lipid-lowering therapy and a low-fat diet. Also, individuals with secondary hypertriglyceridemia (alcohol, unstable diabetes, and medication associated) were excluded.^6^ Individuals with MCS respond better to conventional lipid-lowering therapy, lifestyle modifications, management of secondary factors, and a low-fat diet, and this might explain the better detection rate when stringent selection criteria are used.^1^^,^^9^^,^^15^ By defining FCS phenotype by *LPL* activity of <30%, Surendran et al^32^ found 51% of FCS phenotype to be harboring LOF *LPL* variants, whereas only 16% of the individuals with FCS phenotype had LOF variants in other FCS-causing genes. Interestingly 21% had only a common single-nucleotide polymorphism (SNP) in *LPL* or *APOA5*, whereas no genetic variant was found in 16% of the participants.^32^ A higher detection rate for FCS was found in females (15.4%) compared with males (9.2%), and the mean age at the time of genetic testing for the whole cohort was younger in females. Women tend to have a lower threshold for utilizing health care services, seeking both physical and mental health support more readily than men.^33^, ^34^, ^35^ This proactive approach may have led to earlier age at genetic testing and higher detection rates.

A critical finding from our study is that most individuals with FCS receive their diagnosis in the third or fourth decade of life. Moreover, fewer than 10% of individuals obtain a molecular diagnosis before the age of 20. Our previous research has highlighted a significant delay between the onset of symptoms and the clinical diagnosis of CS in a subset of this cohort, indicating a substantial gap and unmet clinical need.^9^ With the advent of specific drug therapies for FCS^36^ and the anticipated introduction of additional treatments,^15^ it is crucial to prevent delays in molecular diagnosis, which is often necessary for eligibility for disease-modifying therapies. Early diagnosis, particularly in neonates at high risk—such as those from ethnic minorities, offspring of consanguineous unions, those with a family history of hypertriglyceridemia, or the presence of milky-looking plasma—would help to avoid delays in therapeutic interventions aimed at reducing chylomicronemia and associated complications.

Although current genetic testing methods used by both national laboratories in the United Kingdom can exclude a familial cause of severe hypertriglyceridemia, ie, FCS, if no biallelic P/LP variants are identified, the possibility of polygenic hypertriglyceridemia remains. Many cases of chylomicronemia are, in fact, polygenic, arising from the combined effects of multiple genetic variants, which together increase the likelihood of developing chylomicronemia and hypertriglyceridemia.^13^, ^14^, ^15^ Individuals who carry both rare heterozygous P/LP variants and high polygenic scores exhibit significantly elevated triglyceride levels and face a significantly higher risk of acute pancreatitis compared with those without these pathogenic variants and with low polygenic scores. Indeed, the risk of developing severe hypertriglyceridemia and pancreatitis is influenced by polygenic risk score even in the presence of P/LP variant in 1 of the 5 canonical genes in heterozygous state.^37^ An inherent limitation of the current genetic testing methodologies is their focus on coding and proximal intronic regions, which results in the underexploration of deep intronic variants. These deep intronic regions may harbor causative variants that remain undetected by standard NGS. Consequently, testing may exclude a monogenic cause of severe hypertriglyceridemia in the absence of biallelic pathogenic/likely pathogenic (P/LP) variants, failing to account for the potential contribution of deep intronic variants. Additionally, polygenic hypertriglyceridemia represents an emerging area of research. Polygenic risk score is not yet routinely incorporated into clinical practice in United Kingdom for severe hypertriglyceridemia, despite some accumulating evidence from recent past suggesting that a substantial proportion of monogenic-negative individuals may exhibit a polygenic etiology,^38^^,^^39^ that may affect their risk of developing pancreatic complications.^14^^,^^15^^,^^37^ In the United Kingdom, efforts are underway to explore this avenue, with the aim of advancing genomic services for this population.

Several limitations of this study should be considered. First, the novel variants in *LPL*, *GPIHBP1*, *APOA5*, *LMF1*, and *APOC2* identified in this cohort were not functionally assessed; however, we have ascertained their pathogenicity rigorously based on existing genomic databases. We included individuals only who had genetic testing done in the United Kingdom, which might exclude a small minority whose genetic testing was done as part of clinical trials or those who had genetic testing performed overseas; however, the proportion of those would be small. Although identifying index cases is crucial for understanding the diagnostic yield in genetic testing, the absence of this information in our data set represents a limitation. Some positive diagnoses might have originated from the same family. However, there is no standardized protocol for cascade genetic testing within the health care system that this study covers, and family screening is not routinely offered. Future studies should aim to incorporate more comprehensive clinical information, including the identification of index cases and routine cascade testing. Such data will enable a more thorough evaluation of genetic diagnosis outcomes and familial risk management. Although the study provides insights into the prevalence and genetic spectrum of FCS, the clinical outcomes, treatment responses, and long-term prognosis of affected individuals remain beyond the scope of the current investigation, highlighting the need for future research and prospective studies.

This study presented a comprehensive genetic spectrum of FCS-causing genes in the United Kingdom. The data set, comprising genetic analysis from accredited laboratories, represents the largest and most novel collection of FCS and variant-positive MCS cases to date. By encompassing individuals from multiple regional lipid centers and national genetics laboratories, the study offers a robust representation of the genetic landscape of FCS in the United Kingdom. The study’s thorough examination of FCS prevalence, genetic spectrum, geographical distribution, and demographic factors, such as gender and ethnicity, provide valuable insights into the epidemiology of this rare disorder.

### Conclusion

Molecular diagnosis of FCS is often delayed. The genetic architecture of FCS in the United Kingdom is complex, with a significant portion of the cohort affected by P/LP variants in non-*LPL* FCS-causing genes. Non-*LPL*-FCS variants are more common in the UK cohort compared with other cohorts reported in the literature and are more common in individuals from non-European geographic ancestry. This genetic diversity may have important implications for diagnosis, management, and potential therapeutic approaches.

## Data Availability

The data that support the findings of this study are available from the corresponding author upon request.

## Conflict of Interest

Handrean Soran: received personal fees from Amgen, Akcea, Synageva, NAPP, Novartis, Takeda, Sanofi, Pfizer, and Kowa and research grants and donations from Akcea, Pfizer, MSD, Amgen, Genzyme-Sanofi, Synageva, Amryt, Synageva, and Alexion.

Anthony S. Wierzbicki: site investigator on trials from Akcea and Regeneron, Royalties from Elsevier for a book on FCS, and is a board member for familial hyperlipidemia group, Europe.

Dev Datta: received honoraria for advisory boards from SOBI.

Natalie Forrester: received honoraria for presentations from SOBI.

Yee Teoh: received speakers fee from Daiichi-Sankyo and Amarin.

Paul Downie: received Speaker/Consulting fees from Besins Healthcare, Daiichi-Sankyo, Sanofi, Amgen, Sobi, Novartis, and Amarin. Received financial support for travel and accommodation to attend national/international conferences from Sanofi, Amgen, and Daichii-Sanko

Robert A. Hegele: received consulting fees from Acasti, Aegerion, Akcea/Ionis, Amgen, Arrowhead, HLS Therapeutics, Pfizer, Novartis, Regeneron, Sanofi, and UltraGenyx.

All other authors declare no conflicts of interest.
