# Supplementary material for: Rare variant genetic landscape of familial chylomicronemia syndrome (FCS) in the United Kingdom
Source: Genet Med Open. 2025 Jul 14;3:103445. doi: 10.1016/j.gimo.2025.103445 (PMC12409453; doi:10.1016/j.gimo.2025.103445)
Supplement: Supplementary Material [file mmc1.pdf]

Supplementary Information

Rare Variant Genetic Landscape of Familial Chylomicronemia Syndrome (FCS) in the United Kingdom

Bilal Bashir[1,2], Natalie Forrester[3], Paul Downie[4], Sarah Marsh[3], Carolyn Dent[3], Anthony S. Wierzbicki[5], Charlotte Dawson[6], Jonathan Schofield[2], Fiona Jenkinson[7], Michael Mansfield[8], Dev Datta[9], Hannah Delaney[10], Yee Teoh[11], Paul Hamilton[12], Jaimini Cegla[13], Maryam Ferdousi[1], Anoushka Kamath[1], Pankaj Gupta[14], Ahai Luvai[7], Dawn O'Sullivan[15, 16], Deanna Mamood[17], Jian Wang[18], Paul N Durrington[1], Robert A. Hegele[18] and Handrean Soran[1, 2]

1. Faculty of Biology Medicine & Health, University of Manchester, UK
2. Department of Endocrinology, Diabetes and Metabolism, Manchester University NHS Foundation Trust, Manchester, UK.
3. Bristol Genetics Laboratory, North Bristol NHS Trust, Bristol, UK
4. Department of Clinical Biochemistry, Bristol Royal Infirmary, Bristol, UK; Salisbury NHS Foundation Trust, Salisbury UK
5. Department of Metabolic Medicine and Chemical Pathology, Guy's and St. Thomas' Hospitals, London, UK.
6. Department of Diabetes, Endocrinology and Metabolism, Queen Elizabeth Hospital NHS Foundation Trust, Birmingham, UK.
7. Department of Clinical Biochemistry and Metabolic Medicine, Royal Victoria Infirmary, Newcastle upon Tyne, New Castle, UK.
8. Leeds Centre for Diabetes & Endocrinology, Leeds Teaching Hospitals NHS Trust, Leeds, UK
9. Lipid Unit, University Hospital Llandough, Cardiff, UK
10. Department of Clinical Chemistry, Sheffield Teaching Hospitals NHS Foundation Trust, Sheffield, UK.
11. Department of Chemical Pathology & Metabolic Medicine, Wrexham Maelor Hospital, Wrexham, UK
12. Centre for Medical Education, Queen's University Belfast, Belfast BT7 1NN, UK.
13. Division of Diabetes, Endocrinology and Metabolism, Imperial College London, London, UK.
14. University Hospitals of Leicester NHS Trust.
15. North of Scotland Genetics Laboratory, Polwarth Building, Aberdeen, Scotland, UK.
16. Department of Medical Genetics, School of Medicine, Medical Sciences and Nutrition, University of Aberdeen, Aberdeen, AB25 2ZD, UK.
17. College of Medicine, University of Sulaimani, Sulaimani, Federal Region of Kurdistan, Iraq.
18. Robarts Research Institute, Western University. London, Ontario, Canada N6A5B7.

Correspondence:
Professor Handrean Soran MSc, MD, FRCP
Consultant Physician and Endocrinologist, Department of Endocrinology, Diabetes and Metabolism, Manchester University NHS Foundation Trust, Peter Mount Building, 136 Hathersage Rd, Manchester M13 0HY, United Kingdom.
E-mail: hsoran@aol.com
Secretary Tel: +44 (0) 161 276 4066, Fax: +44 (0) 161 276 3630

Supplemental Table 1: Distribution of individuals tested for FCS by age group

Age Group (years)	Diagnosis		
	**FCS n=114**	**P/LP Variant Positive MCS n=100**	**P/LP Variant Negative MCS n=666**
<1	7 (6.1)	1 (1)	4 (0.6)
1-10	8 (7.0)	2 (1)	15 (2.2)
10-20	11 (9.6)	7 (7)	28 (4.2)
20-30	17 (14.9)	11 (11)	41 (6.1)
30-40	24 (21.0)	27 (27)	144 (21.6)
40-50	27 (23.7)	24 (24)	203 (30.5)
50-60	8 (7.0)	15 (15)	160 (24.0)
60-70	9 (7.9)	9 (9)	62 (9.3)
>70	3 (2.6)	4(4)	9(1.3)

FCS: Familial chylomicronemia syndrome; MCS: Multifactorial chylomicronemia syndrome

Supplemental Table 2: Lipoprotein Lipase (LPL) gene variants identified in the UK

Zygosity	HGVS c.description	Exon	HGVS p.description	HGVS g.description	Variant type	ACMG Classification			No. of individuals FCS/MCS[a]
						Inhouse database	Franklin	Varsome	
LPL									
Homozygous	c.88+1G>C	1	p.?	NC_000008.10:g.19797040G>C	Splice site	P	P	LP	1
	**c.112G>T**	**3**	**p.(Glu38*)**	**NC_000008.10:g.19805714G>T**	**Nonsense**	**P**	**LP**	**LP**	**1**
	c.273G>A	3	p.(Trp91*)	NC_000008.10:g.19809303G>A	Nonsense	P	LP	LP	1
	c.289_294delinsTTTGCCAAAA	3	p.(Ala97Phefs*52)	NC_000008.10:g.19809319_19809324delinsTTTGCCAAAA	Frameshift	P	P	P	1
	c.337T>G	3	p.(Trp113Gly)	NC_000008.10:g.19809367T>G	Missense	LP	LP	LP	1
	**c.384delinsTGGGCT**	**3**	**p.(Lys129Glyfs*45)**	**NC_000008.10:g.19809414delinsTGGGCT**	**Frameshift**	**P**	**LP**	**LP**	**1**
	c.482G>A	4	p.(Gly161Glu)	NC_000008.10:g.19810873G>A	Missense	LP	VUS	LP	1
	**c.496_497del**	**4**	**p.(Gly166Hisfs*8)**	**NC_000008.10:g.19810887_19810888del**	**Frameshift**	**P**	**LP**	**LP**	**1**
	c.553G>A	5	p.(Ala185Thr)	NC_000008.10:g.19811642G>A	Missense	P	LP	LP	6
	c.644G>A	5	p.(Gly215Glu)	NC_000008.10:g.19811733G>A	Missense	P	P	P	10
	c.679G>T	5	p.(Val227Phe)	NC_000008.10:g.19811768G>T	Missense	LP	LP	P	1
	c.680T>C	5	p.(Val227Ala)	NC_000008.10:g.19811769T>C	Missense	LP	LP	P	1
	c.721C>T	5	p.(Pro241Ser)	NC_000008.10:g.19811810C>T	Missense	LP	P	LP	1
	c.784C>T	6	p.(Gln262*)	NC_000008.10:g.19813360C>T	Nonsense	P	P	P	3
	c.809G>A	6	p.(Arg270His)	NC_000008.10:g.19813385G>A	Missense	P	P	P	1
	c.829G>A	6	p.(Asp277Asn)	NC_000008.10:g.19813405G>A	Missense	LP	P	P	1
	c.835C>G	6	p.(Leu279Val)	NC_000008.10:g.19813411C>G	Missense	P	P	P	1
	**c.986A>G**	**6**	**p.(Tyr329Cys)**	**NC_000008.10:g.19813562A>G**	**Missense**	**LP**	**LP**	**LP**	**2**
	c.989T>C	6	p.(Leu330Pro)	NC_000008.10:g.19813565T>C	Missense	LP	LP	LP	2
	c.998G>A	6	p.(Arg333His)	NC_000008.10:g.19813574G>A	Missense	LP	P	LP	1
	Whole gene deletion	-	-	NC_000008.10:g.?del	Deletion	P	P	P	3

	**c.49_50del**	**1**	**p.(Leu18Aspfs*22)**	**NC_000008.10:g.19797000_19797001del**	**Frameshift**	**P**	**LP**	**LP**	**0/1**
	c.88+2dupT	1	p.?	NC_000008.10:g.19797041dup	Splice site	P	LP	VUS	1/0
	**c.88+5G>C**	**1**	**p.?**	**NC_000008.10:g.19797044G>C**	**Splice site**	**LP**	**VUS**	**VUS**	**1/0**
	c.88+2T>G	1	p.?	NC_000008.10:g.19797041T>G	Splice site	P	LP	LP	0/1
	c.89-1G>A	1	p.?	NC_000008.10:g.19805690G>A	Splice site	P	P	P	0/1
	Exon 2-10 deletion	2-10		NC_000008.10:g.(?_19798663)_(19829240_?)del	Deletion	P			0/1
	**c.133_143del**	**2**	**p.(Thr45Hisfs*3)**	**NC_000008.10:g.19805735_19805745del**	**Frameshift**	**P**	**P**	**LP**	**0/1**
	c.209A>G	2	p.(Asn70Ser)	NC_000008.10:g.19805811A>G	Missense	P	VUS	LP	0/1
	c.286G>C	3	p.(Val96Leu)	NC_000008.10:g.19809316G>C	Missense	P	P	LP	1/0
**Heterozygous**	c.292G>A	3	p.(Ala98Thr)	NC_000008.10:g.19809322G>A	Missense	LP	P	LP	2/2
	**c.339_342delGCTG**	**3**	**p.(Trp113Cysfs*58)**	**NC_000008.10:g.19809369_19809372del**	**Frameshift**	**P**	**LP**	**LP**	**0/2**
	**c.344C>G**	**3**	**p.(Ser115*)**	**NC_000008.10:g.19809374C>G**	**Nonsense**	**P**	**LP**	**LP**	**2/0**
	c.397C>T	3	p.(Gln133*)	NC_000008.10:g.19809427C>T	Nonsense	P	P	P	3/0
	c.440_443del	4	p.(Asn147Thrfs*24)	NC_000008.10:g.19810831_19810834del	Frameshift	P	P	P	2/1
	**c.466_467del**	**4**	**p.(Leu156Glyfs*18)**	**NC_000008.10:g.19810857_19810858del**	**Frameshift**	**P**	**LP**	**LP**	**0/1**
	c.474C>G	4	p.(Tyr158*)	NC_000008.10:g.19810865C>G	Nonsense	P	P	P	0/2
	c.553G>A	5	p.(Ala185Thr)	NC_000008.10:g.19811642G>A	Missense	P	LP	LP	0/1
	c.566_567del	5	p.(Phe189*)	NC_000008.10:g.19811655_19811656del	Nonsense	P	LP	P	0/1
	c.630C>G	5	p.(His210Gln)	NC_000008.10:g.19811719C>G	Missense	LP	LP	LP	1/0
	**c.641G>C**	**5**	**p.(Arg214Thr)**	**NC_000008.10:g.19811730G>C**	**Missense**	**LP**	**LP**	**LP**	**1/0**
	c.644G>A	5	p.(Gly215Glu)	NC_000008.10:g.19811733G>A	Missense	P	P	P	15/23
	c.662T>C	5	p.(Ile221Thr)	NC_000008.10:g.19811751T>C	Missense	P	P	P	1/2
	c.701C>T	5	p.(Pro234Leu)	NC_000008.10:g.19811790C>T	Missense	P	P	P	0/2
	c.721C>T	5	p.(Pro241Ser)	NC_000008.10:g.19811810C>T	Missense	LP	P	LP	2/0
	c.727T>A	5	p.(Cys243Ser)	NC_000008.10:g.19811816T>A	Missense	LP	LP	LP	0/1

	**c.775+1G>A**	**5**	**p.?**	**NC_000008.10:g.19811865G>A**	**Splice site**	**P**	**LP**	**LP**	**0/2**
	c.784C>T	6	p.(Gln262*)	NC_000008.10:g.19813360C>T	Missense	P	P	P	0/2
	c.808C>T	6	p.(Arg270Cys)	NC_000008.10:g.19813384C>T	Missense	LP	P	P	0/1
	c.809G>A	6	p.(Arg270His)	NC_000008.10:g.19813385G>A	Missense	P	P	P	2/1
	c.835C>G	6	p.(Leu279Val)	NC_000008.10:g.19813411C>G	Missense	LP	P	P	0/1
	c.940G>A	6	p.(Gly314Ser	NC_000008.10:g.19813516G>A	Missense	LP	LP	VUS	0/1
	**c.986A>G**	**6**	**p.(Tyr329Cys)**	**NC_000008.10:g.19813562A>G**	**Missense**	**LP**	**LP**	**LP**	**1/0**
	c.1018G>A	6	p.(Val340Ile)	NC_000008.10:g.19813594G>A	Missense	LP	VUS	LP	2/2
	Partial exon 6 dup	6		NC_000008.10:g.(19813474)_(19813794_?)dup	Duplication	P	P	P	1/2
	c.1262G>A	8	p.(Trp421*)	NC_000008.10:g.19818534G>A	Non-sense	P	LP	LP	0/1
	Whole gene deletion	-	-	NC_000008.10:g.19798663_19829240del	Deletion	P	P	P	3/0

[a]Number against homozygous row corresponds only to the number of FCS cases
Variants reported in bold are novel variants that are not reported before
HGVS c. descriptions are based on transcript reference sequence NM_000237.3 for the LPL gene.
LP: Likely Pathogenic, P: Pathogenic, VUS: Variant of Uncertain Significance

Supplemental Table 3: non-Lipoprotein Lipase (non-LPL) gene variants identified in the UK

**Zygosity**	**HGVS c. description**	**Exon**	**HGVS p.description**	**HGVS g.description**	**Variant type**	**ACMG Classification**			**No. of individuals FCS/MCS[a]**
						**Inhouse database**	**Franklin**	**Varsome**	
APOA5									
	c.16_39del	1	p.(Ala6_Ala13del)	NC_000011.9:g.116662543_116662566del	Missense	LP	LP	VUS	1
	**c.77del**	**2**	**p.(Gly26Alafs*31)**	**NC_000011.9:g.116662387del**	**Frameshift**	**P**	**LP**	**LP**	**1**
	**c.205A>T**	**3**	**p.(Lys69*)**	**NC_000011.9:g.116661740T>A**	**Nonsense**	**P**	**LP**	**LP**	**1**
	c.289C>T	3	p.(Gln97*)	NC_000011.9:g.116661656G>A	Nonsense	P	P	P	2
**Homozygous**	c.427del	3	p.(Arg143Alafs*57)	NC_000011.9:g.116661518del	Frameshift	P	P	P	5
	**c.483del**	**3**	**p.(Gln161Hisfs*39)**	**NC_000011.9:g.116661462del**	**Frameshift**	**P**	**LP**	**LP**	**2**
	c.16_39del	1	p.(Ala6_Ala13d)	NC_000011.9:g.116662543_116662566del	Missense	LP	LP	VUS	0/2
	c.161+5G>C	2	p.?	NC_000011.9:g.116662297C>G	Splice site	LP	VUS	LP	3/5
	c.289C>T	3	p.(Gln97*)	NC_000011.9:g.116661656G>A	Nonsense	P	P	P	5/12
	c.427del	3	p.(Arg143Alafs*57)	NC_000011.9:g.116661518del	Frameshift	P	P	P	0/6
**Heterozygous**	**c.617_632del**	**3**	**p.(Val206Alafs*86)**	**NC_000011.9:g.116661315_116661330del**	**Frameshift**	**P**	**LP**	**LP**	**0/1**
	c.758T>C	3	p.(Leu253Pro)	NC_000011.9:g.116661187A>G	Missense	LP	VUS	LP	0/1
	c.823C>T	3	p.(Gln275*)	NC_000011.9:g.116661122G>A	Nonsense	P	P	P	2/10
GPIHBP1									
	**c.53-1G>A**	**2**	**p.?**	**NC_000008.10:g.144295696G>A**	**Splice site**	**P**	**LP**	**LP**	**1**
	c.239C>A	3	p.(Thr80Lys)	NC_000008.10:g.144296945C>A	Missense	LP	VUS	VUS	1
**Homozygous**	c.323C>T	4	p.(Thr108Met)	NC_000008.10:g.144297161C>T	Missense	LP	VUS	VUS	4
	c.394C>T	4	p.(Gln132*)	NC_000008.10:g.144297232C>T	Nonsense	P	LP	LP	1
	Exon 3-4 del	3-4	-	NC_000008.10:g.(?_144296868)_(144298897_?)del	Multiexon deletion	P	P	P	15
**Heterozygous**	c.394C>T	4	p.(Gln132*)	NC_000008.10:g.144297232C>T	Nonsense	P	LP	LP	0/1
LMF1									
	c.1024C>T	7	p.(Arg342*)	NC_000016.9:g.921215G>A	Nonsense	P	P	P	3
	**c.1051C>T**	**7**	**p.(Arg351*)**	**NC_000016.9:g.921188G>A**	**nonsense**	**P**	**P**	**LP**	**1**
**Homozygous**	**c.1264C>T**	**9**	**p.(Gln422*)**	**NC_000016.9:g.920035G>A**	**Nonsense**	**P**	**P**	**P**	**3**

**Heterozygous**	c.244_245del	2	p.(Arg82Glyfs*81)	NC_000016.9:g.1004615_1004616del	Frameshift	P	P	P	0/1
	c.1348A>T		p.(Arg450*)	NC_000016.9:g.919951T>A	Nonsense	P	LP	LP	1/0
APOC2									
**Homozygous**	**c.215+2del**	**3**	**p.?**	**NC_000019.9:g.45452119del**	**Splice site**	**P**	**LP**	**LP**	**2**
	c.215G>C	3	p.(Arg72Thr)	NC_000019.9:g.45452117G>C	Missense	LP	VUS	P	2
	c.281T>C	4	p.(Leu94Pro)	NC_000019.9:g.45452481T>C	Missense	LP	VUS	VUS	1
	c.274dupC	4	p.(Gln92Profs*28)	NC_000019.9:g.45452474dup	Frameshift	P	LP	LP	1

[a]Number against homozygous row corresponds only to the number of FCS cases

Variants reported in bold are novel variants that are not reported before

HGVS c. descriptions are based on transcript reference sequence NM_052968.5 for the *APOA5*, NM_022773.4 for *LMF1*, NM_178172.6 for *GPIHBP1* and for NM_000483.5 *APOC2* gene.

LP: Likely Pathogenic, P: Pathogenic, VUS: Variant of Uncertain Significance

Supplemental Table 4: Variants of uncertain significance (VUS) identified in UK in FCS causing genes

Zygosity	HGVS c. description	Exon	HGVS p. description	HGVS g. description	Variant type	ACMG Classification			No. of individuals
						Inhouse database	Franklin	Varsome	
				LPL					
**Homozygous**	**c.371C>A**	**3**	**p.(Ser124Tyr)**	**NC_000008.10:g.19809401C>A**	**Missense**	**VUS**	**VUS**	**LP**	**1**
	**c.425T>C**	**3**	**p.(Met142Thr)**	**NC_000008.10:g.19809455T>C**	**Missense**	**VUS**	**VUS**	**VUS**	**1**
	**c.567T>G**	**5**	**p.(Phe189Leu)**	**NC_000008.10:g.19811656T>G**	**Missense**	**VUS**	**LP**	**LP**	**1**
	c.953A>G	6	p.(Asn318Ser)	NC_000008.10:g.19813529A>G	Missense	VUS	VUS	B	3
**Heterozygous**	**c.325A>T**	**3**	**p.(ile109Phe)**	NC_000008.10:g.19809355A>T	**Missense**	**VUS**	**VUS**	**VUS**	**1**
	c.326T>C	3	p.(Ile109Thr)	NC_000008.10:g.19809356T>C	Missense	VUS	VUS	LP	1
	**c.332_334delTGG**	**3**	**p.(Val111del)**	**NC_000008.10:g.19809362_19809364del**	**In-frame deletion**	**VUS**	**VUS**	**LP**	**1**
	c.347G>C	3	p.(Arg116Pro)	NC_000008.10:g.19809377G>C	Missense	VUS	LP	LP	1
	c.464T>C	4	p.(Leu155Pro)	NC_000008.10:g.19810855T>C	Missense	VUS	LP	LP	1
	**c.590G>C**	**5**	**p.(Arg197Pro)**	**NC_000008.10:g.19811679G>C**	**Missense**	**VUS**	**LP**	**P**	**1**
	c.953A>G	6	p.(Asn318Ser)	NC_000008.10:g.19813529A>G	Missense	VUS	VUS	B	14
	c.1139+7A>G	7	p.?	NC_000008.10:g.19816898A>G	Splice site	VUS	LP	VUS	1
	**c.1186G>C**	**8**	**p.(Glu396Gln)**	**NC_000008.10:g.19818458G>C**	**Missense**	**VUS**	**VUS**	**LP**	**3**
	**c.1205T>G**	**8**	**p.(Leu402Arg)**	**NC_000008.10:g.19818477T>G**	**Missense**	**VUS**	**VUS**	**LP**	**1**
	**c.1307G>T**	**8**	**p.(Gly436Val)**	**NC_000008.10:g.19818579G>T**	**Missense**	**VUS**	**LP**	**LP**	**1**
				APOA5					
**Homozygous**	c.553G>T	3	p.(Gly185Cys)	NC_000011.9:g.116661392C>A	Missense	VUS	VUS	B	1
**Heterozygous**	**c.287_289del**	**3**	**p.(Leu96del)**	**NC_000011.9:g.116661658_116661660del**	**In-frame deletion**	**VUS**	**VUS**	**VUS**	**1**
	**c.316C>T**	**3**	**p.(Arg106Cys)**	**NC_000011.9:g.116661629G>A**	**Missense**	**VUS**	**VUS**	**VUS**	**2**
	**c.353T>G**	**3**	**p.(val118Gly)**	**NC_000011.9:g.116661592A>C**	**Missense**	**VUS**	**VUS**	**VUS**	**3**
	c.434A>G	3	p.(Gln145Arg)	NC_000011.9:g.116661511T>C	Missense	VUS	VUS	LB	1
	c.553G>T	3	p.(Gly185Cys)	NC_000011.9:g.116661392C>A	Missense	VUS	VUS	B	9
	**c.610C>T**	**3**	**p.(Arg204Cys)**	**NC_000011.9:g.116661335G>A**	**Missense**	**VUS**	**VUS**	**VUS**	**1**
	**c.631C>T**	**3**	**p.(Arg211Cys)**	**NC_000011.9:g.116661314G>A**	**Missense**	**VUS**	**VUS**	**VUS**	**1**
	c.644C>T	3	p.(Pro215Leu)	NC_000011.9:g.116661301G>A	Missense	VUS	LB	LB	1

	**c.720_764del45**	**3**	**p.(Ala241_glu255 del)**	**NC_000011.9:g.116661182_116661226del**	**In-frame deletion**	**VUS**	**VUS**	**VUS**	**1**
	c.944C>T	3	p.(Ala315Val)	NC_000011.9:g.116661001G>A	Missense	VUS	VUS	B	1
	**c.968C>T**	**3**	**p.(Ala323Val)**	**NC_000011.9:g.116660977G>A**	**Missense**	**VUS**	**VUS**	**LB**	**1**
LMF1									
**Homozygous**	**c.755C>T**	**6**	**p.(Ala252Val)**	**NC_000016.9:g.929712G>A**	**Missense**	**VUS**	**VUS**	**VUS**	**1**
	c.1247G>A	9	p.(Arg416Gln)	NC_000016.9:g.920052C>T	Missense	VUS	VUS	LP	2
	c.1609C>T	11	p.(Arg537Trp)	NC_000016.9:g.904627G>A	Missense	VUS	VUS	VUS	1
	c.1621G>A	11	p.(Gly541Arg)	NC_000016.9:g.904615C>T	Missense	VUS	LB	LB	1
**Heterozygous**	**c.104G>A**	**1**	**p.(Arg35His)**	**NC_000016.9:g.1020877C>T**	**Missense**	**VUS**	**VUS**	**LB**	**1**
	**c.112G>A**	**1**	**p.(Ala38Thr)**	**NC_000016.9:g.1020869C>T**	**Missense**	**VUS**	**VUS**	**LB**	**1**
	c.683G>A	5	p.(Gly228Glu)	NC_000016.9:g.943053C>T	Missense	VUS	VUS	VUS	2
	c.683G>T	5	p.(Gly228Val)	NC_000016.9:g.943053C>A	Missense	VUS	VUS	VUS	1
	c.787C>T	6	p.(His263Tyr)	NC_000016.9:g.929680G>A	Missense	VUS	VUS	VUS	1
	**c.805A>T**	**6**	**p.(Ser269Cys)**	**NC_000016.9:g.929662T>A**	**Missense**	**VUS**	**VUS**	**LB**	**1**
	**c.1328G>A**	**9**	**p.(Cys443Tyr)**	**NC_000016.9:g.919971C>T**	**Missense**	**VUS**	**VUS**	**VUS**	**1**
	c.1351C>T	9	p.(Arg451Trp)	NC_000016.9:g.919948G>A	Missense	VUS	B	B	1
	**c.1405G>A**	**9**	**p.(Ala469Thr)**	**NC_000016.9:g.919894C>T**	**Missense**	**VUS**	**LB**	**LB**	**1**
	**c.1628_1646del**	**11**	**p.(Tyr543Trpfs*4)**	**NC_000016.9:g.904595_904613del**	**Frameshift**	**VUS**	**VUS**	**VUS**	**1**
	**c.1688T>G**	**11**	**p.(Leu563Arg)**	**NC_000016.9:g.904548A>C**	**Missense**	**VUS**	**LB**	**LB**	**1**
GBIHBP1									
**Homozygous**	**c.188T>C**	**3**	**p.(Leu63Pro)**	**NC_000008.10:g.144296894T>C**	**Missense**	**VUS**	**VUS**	**VUS**	**1**
**Heterozygous**	**c.52+28G>A**	**1**	**p.?**	**NC_000008.10:g.144295222G>A**	**Splice site**	**VUS**	**VUS**	**LB**	**1**
	**c.416C>T**	**4**	**p.(Pro139Leu)**	**NC_000008.10:g.144297254C>T**	**Missense**	**VUS**	**VUS**	**VUS**	**1**
	**c.484G>A**	**4**	**p.(Glu162Lys)**	**NC_000008.10:g.144297322G>A**	**Missense**	**VUS**	**VUS**	**LB**	**1**

Variants reported in bold are novel variants that are not reported before

HGVS c. descriptions are based on transcript reference sequence NM_052968.5 for the *APOA5*, NM_022773.4 for *LMF1*, NM_178172.6 for *GPIHBP1* and for NM_000483.5 *APOC2* gene.

B: Benign, LB: Likely benign, LP: Likely Pathogenic, P: Pathogenic, VUS: Variant of Uncertain Significance

Supplemental Table 5: Geographical distribution of FCS testing and detection rate

Region	Total population (millions)	No. of tests for FCS done	No. of tests/ million population	FCS	No. of FCS cases/million	Detection rate
London	8.9	179	20.0	29	3.3	16.2
North-west	7.5	109	14.5	32	4.3	29.3
West Midlands	4.9	86	17.4	16	3.2	18.6
East of England	6.4	24	3.7	3	0.5	12.5
East Midlands	6.0	77	12.8	2	0.3	2.6
Northeast	2.7	24	8.9	3	1.1	12.5
Southwest	5.81	89	15.4	3	0.5	3.4
Southeast	9.4	48	5.1	8	0.9	16.7
Yorkshire & Humber	5.5	37	6.7	9	1.6	24.3
Scotland	5.4	168	30.8	5	0.9	3.0
Wales	3.1	27	8.6	2	0.6	7.4
Northern Ireland	1.9	12	6.3	2	1.0	16.7

Supplemental Figure 1: Molecular changes

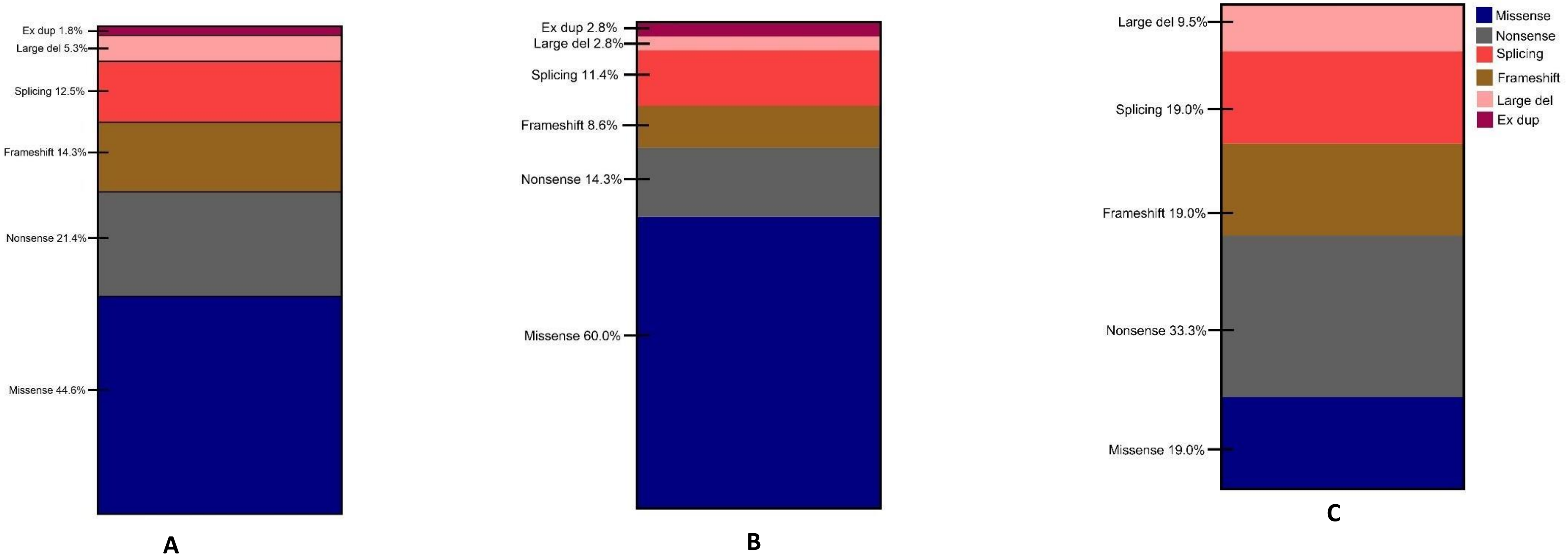

Supplemental Figure 1: Molecular changes in variants of FCS causing genes in whole cohort (A) *LPL*-FCS (B) non-*LPL*-FCS (C)
